# Comparative Genomics Analysis Provides New Strategies for Bacteriostatic Ability of *Bacillus velezensis* HAB-2

**DOI:** 10.3389/fmicb.2020.594079

**Published:** 2020-11-17

**Authors:** Peidong Xu, Shangqian Xie, Wenbo Liu, Pengfei Jin, Dandan Wei, Dahar Ghulam Yaseen, Yu Wang, Weiguo Miao

**Affiliations:** ^1^Key Laboratory of Green Prevention and Control of Tropical Plant Diseases and Pests, Ministry of Education, College of Plant Protection, Hainan University, Haikou, China; ^2^School of Life and Pharmaceutical Sciences, Hainan University, Haikou, China; ^3^College of Forestry, Hainan University, Haikou, China

**Keywords:** *Bacillus velezensis*, genome, secondary metabolites, prophage, 4′-phosphopantetheinyl transferases

## Abstract

Biocontrol formulations prepared from biocontrol bacteria are increasingly applied in sustainable agriculture. Notably, inoculants prepared from *Bacillus* strains have been proven efficient and environmentally friendly alternatives to chemical bactericides. The bacterium *Bacillus velezensis* HAB-2 (formerly classified as *B. amyloliquefaciens* HAB-2) is used as a biological control agent in agricultural fields. In this study, we reported a high-quality genome sequence of HAB-2 using third-generation sequencing technology (PacBio RS II). The 3.89 Mb genome encoded 3,820 predicted genes. Comparative analysis among the genome sequences of reference strains *B. velezensis* FZB42, *B. amyloliquefaciens* DSM7 and *B. subtilis* 168 with the HAB-2 genome revealed obvious differences in the variable part of the genomes, while the core genome shared by FZB42 and HAB-2 was similar (96.14%). However, there were differences in the prophage region among the four strains. The numbers of prophage regions and coding genes in HAB-2 and FZB42 were smaller than the other two strains. The HAB-2 genome showed superior ability to produce secondary metabolites and harbored 13 gene clusters involved in synthesis of antifungal and antibacterial acting secondary metabolites. Furthermore, there were two unique clusters: one cluster which encoded lanthipeptide was involved in mersacidin synthesis and another cluster which encoded ladderane was shown to direct an unknown compound. Multidomain enzymes, such as non-ribosomal peptide synthetase and polyketide synthase, control the biosynthesis of secondary metabolites and rely on 4′-phosphopantetheinyl transferases (PPTases). Key genes *lpaH2* and *a* encoded PPTases in HAB-2 encoded 224 and 120 amino acids, respectively. The genomic features revealed that HAB-2 possesses immense potential to synthesize antimicrobial acting secondary metabolites by regulating and controlling gene clusters. The prophage regions and genes encoding PPTases may provide novel insight for the bacteriostatic mechanism of *Bacillus* in the biological control of plant diseases.

## Introduction

Pathogenic microorganisms are a major and chronic threat to food production and ecosystem stability all over the world by infecting plant tissue and affecting plant health (Compant et al., 2005). As the human population has increased and agricultural production has intensified over the past few decades, producers have been increasingly dependent on agrochemicals to control plant diseases (Schäfer and Adams, 2015). However, excessive use of chemical pesticides has had a huge negative impact on the ecological environment. Increasingly, researchers and producers realize the need for alternatives to chemical pesticides to avoid damaging the environment. Use of beneficial bacteria as alternatives to chemical pesticides in plant protection is steadily increasing and is beginning to replace some chemical pesticides (Qiao et al., 2014). In China, according to the China Pesticide Information Network^1^, a total of 41,614 pesticide products were registered in China at the end of May 2020. Among them, 10,971 antiseptics accounted for 26.3%. There were 434 actual microbial pesticides, and registered biological pesticides only accounted for 2.9% of the total pesticide products. Thus, prevention and control of plant diseases are still dominated by chemical pesticides in China, and the road to replace them with biological pesticides is long, but the development potential is huge. As a consequence, researchers are also focusing on the antibacterial mechanisms of biocontrol bacteria.

*Bacillus velezensis* is a biocontrol bacterium that is efficient, environmentally friendly and suitable for wide use for plant protection in agriculture. It produces natural products that are antimicrobial, antiviral and nematocidal (Mariappan et al., 2012). Recently, whole-genome assemblies of *Bacillus* spp. have been produced using high-throughput sequencing, and genome analysis of *B. velezensis* reveals their potential for biocontrol of plant pathogens (Chen et al., 2008). The *B. velezensis* genome harbors some important gene clusters involved in the synthesis of secondary metabolites. The cluster of protective molecules is generally made by non-ribosomal peptide synthetase (NRPS) and polyketide synthase (PKS) (Schneider et al., 2007; Chen et al., 2008; Beld et al., 2014). Five gene clusters (*srf*, *bmy*, *fen*, *nrs*, and *dhb*) were shown to direct synthesis of surfactin, fengycin and iturin (including bacillomycinD and mycosubtilin). Three gene clusters, *mln*(*pks1*), *bae*(*pks2*), and *dfn*(*pks3*), were shown to direct synthesis of the polyketides macrolactin, bacillaene and difficidin, respectively (Koumoutsi et al., 2004). The size of these gene clusters generally accounts for 7.5–8.5% of the genome of the strain (Koumoutsi et al., 2004; Chen et al., 2008).

In *B. velezensis*, NRPS and PKS control the biosynthesis of secondary metabolites (Metz et al., 2001; Cox, 2007). However, these enzymes are inactive until they are post-translationally modified. The activation of apo-peptidyl carrier proteins of NRPS and apo-acyl carrier proteins of PKS to their active holo form is accomplished by dedicated 4′-phosphopantetheinyl transferases (PPTases) (Lambalot et al., 1996). The synthesis of non-ribosomal lipopeptides and polyketides are dependent on the PPTases, which are encoded by a functional *sfp* gene (Chen et al., 2008). A PPTase *sfp* was first identified by Nakano et al. (1990, 1992) in attempts to transform the non-surfactin-producing strain *B. subtilis* JH642. Borchert et al. (1994) identified a gene, *gsp*, with 34% identity and 54% homology to *sfp*. Huang et al. (1993) discovered *lpa-14*, showing homology to *sfp*, which is related to iturin A. Because of their cornerstone role in metabolism, PPTases are considered antibiotic targets in agriculture. Being indispensable for the biosynthesis of natural products with antibiotic action, the PPTase super family genes play a leading role in antibiotic engineering efforts for agriculture.

The bacterium *B. velezensis* HAB-2 has the potential as a biological pesticide due to its excellent antibacterial ability. Our previous study provided evidence that HAB-2 was evaluated against 17 plant pathogens such as *Colletotrichum gloeosporioides*, *Alternaria solani*, and *Fusarium oxysporum*. The inhibition rates were ranged from 42.99–69.76%. Furthermore, the strain could effectively inhibit the infection of *C. gloeosporioides* on the mango leaf and fruits, *Alternaria solani* on the tomato fruits, and prevent and control *Oidium heveae* (Jin et al., 2018). Analysis of the whole genome of *B. velezensis* will allow uncovering the antibacterial mechanism of biocontrol bacteria. In this study, we assembled the whole genome of *B. velezensis* HAB-2, which not only showed a similar ability to produce antimicrobial secondary metabolites as for most *Bacillus* spp., but also demonstrated some unique abilities in our previous study (Jin et al., 2017). Furthermore, the assembled genome of HAB-2 was compared with the genomes of *B. velezensis* FZB42, *B. amyloliquefaciens* DSM7, and *B. subtilis* 168; genome annotation and comparative genomics analysis showed some important features including prophage regions, unique clusters, and key genes of these strains and also revealed that HAB-2 had great potential for biocontrol of plant pathogens.

## Materials and Methods

### Strain Culture and Type Genomes

*Bacillus velezensis* HAB-2 was isolated from cotton (*Gossypium hirsutum*) root-soil in Xinjiang Province, China, and preserved in the Key Laboratory of Green Prevention and Control of Tropical Plant Diseases and Pests (Hainan University), Ministry of Education. The HAB-2 strain was cultivated in Luria–Bertani agar medium and broth at 28°C with shaking at 180 r/min, and was used to extract genomic DNA. Type genome sequences of strains *B. velezensis* FZB42 (Chen et al., 2007, NC_009725.1), *B. amyloliquefaciens* DSM7 (Rückert et al., 2011, NC_014551.1) and *B. subtilis* 168 (Barbe et al., 2009, NC_000964.3) were collected from the National Center for Biotechnology Information (NCBI).

### Genomic DNA Extraction and Sequencing

Genomic DNA from HAB-2 was extracted using commercial kits according to the instructions (Sigma Aldrich, St. Louis, MO, United States). The DNA quality was determined using Qubit and Nanodrop (both Thermo Fisher Scientific, Waltham, MA, United States). Then the qualified genomic DNA from *B. velezensis* HAB-2 was fragmented with G-tubes (Covaris, Woburn, MA, United States) and end-repaired to prepare SMRTbell DNA template libraries (with fragment size > 10 kb selected by bluepippin system) according to the specification (PacBio, Menlo Park, CA, United States). Library quality was detected by Qubit, and average fragment size was estimated on a Bioanalyzer 2100 (Agilent, Santa Clara, CA, United States). The SMRT sequencing was performed on a Pacific Biosciences RSII sequencer (PacBio, Menlo Park, CA, United States) according to standard protocols (MagBead Standard Seq v2 loading, 1 × 180 min movie) using P4-C2 chemistry.

### Genome Assembly

Continuous long reads were generated from three SMRT sequencing runs. Reads > 500 bp with a quality value > 0.75 were merged into a single dataset. Next, the Hierarchical Genome Assembly Process (HGAP) pipeline (Chin et al., 2013) was used to correct for random errors in the long seed reads (seed length threshold 6 kb). The resulting corrected and preassembled reads were used for *de novo* assembly using Celera Assembler with an overlap-layout-consensus strategy (Myers et al., 2000). Since SMRT sequencing features very little variation in quality throughout the reads (Koren et al., 2012), no quality values were used during the assembly. To validate the assembly quality and determine the final genome sequence, the Quiver consensus algorithm (Chin et al., 2013) was used to polish the assembly. Finally, the ends of the assembled sequence were trimmed to circularize the genome.

### Genomic Features Prediction and Annotations

The open reading frame was predicted using GeneMarkS (Besemer et al., 2001). Repetitive elements were identified by RepeatMasker (Tarailo-Graovac and Chen, 2009). Non-coding RNA, such as rRNA, prediction was carried out using rRNAmmer (Lagesen et al., 2007), tRNAs were identified by tNRAscan (Lowe and Eddy, 1997) and compared with the Rfam database, and sRNAs were obtained using Infernal (Gardner et al., 2009). Several complementary approaches were utilized to annotate the assembled sequences. The genes were annotated by aligning with those deposited in diverse protein databases (*E-*value ≤ 1e-5, minimal alignment length percentage ≥ 40%) including the NCBI non-redundant protein (Nr) database, UniProt/Swiss-Prot (UniProt Consortium, 2015), Kyoto Encyclopedia of Genesand Genomes (KEGG) (Kanehisa et al., 2016), Gene Ontology (GO) (Ashburner et al., 2000; Jones et al., 2014), Cluster of Orthologous Groups of proteins (COG) (Galperin et al., 2015; Makarova et al., 2015) and protein families (Pfam) (Finn et al., 2014). Additional annotation was carried out using the following databases: Pathogen Host Interactions (PHI) (Winnenburg et al., 2006), Virulence Factors of Pathogenic Bacteria (VFDB) (Chen et al., 2016), Antibiotic Resistance Genes Database (ARDB) (Liu and Pop, 2009), and Carbohydrate-Active enZYmes (CAZy) (Levasseur et al., 2013). Prophage was predicted using the PHAge SearchTool (PHAST^2^) as described by Zhou et al. (2011). Based on Nr annotation, GO annotation was carried out using Blast2GO (Conesa et al., 2005), and Pfam annotation was applied with PfamScan^3^ (Finn et al., 2014).

### Comparative Genomic Analysis

In order to ensure the same annotation conditions, the genome data of the reference strains are analyzed based on the updated data in 2020 from NCBI, and the genome features are based on NCBI annotation. Genomics comparison of HAB-2, FZB42, DSM7, and 168 was performed using MUMmer (Kurtz et al., 2004) and Mauve (version 2.3.1) (Darling et al., 2010). Gene families were constructed using genes of the reference and the target bacteria, and then the gene families were analyzed using the EDGAR software framework (Blom et al., 2009) to produce a Venn diagram. Phylogenetic analysis was performed to validate the phylogenetic relationship of HAB-2. The phylogenetic tree was constructed by the array of SNPs obtained from alignment of complete sample genomes against the reference. The phylogenetic tree drawn from the genomes of the four strains was constructed by TreeBeST (Nandi et al., 2010) using the method of PHYML (Wang D. et al., 2010) with bootstrap setting as 1,000. Based on 16S rRNA sequences, the phylogenetic tree was constructed using the maximum likelihood method in MEGA7 software (Tamura et al., 2007) with bootstrap setting as 1,000. The secondary metabolite antibacterial gene clusters of the four strains were compared and analyzed using anti-SMASH 4.1.0 (Weber et al., 2015).

## Results

### Distinctive Genomic Features in the Prophage Region of *Bacillus* Strains

**FIGURE 1:**
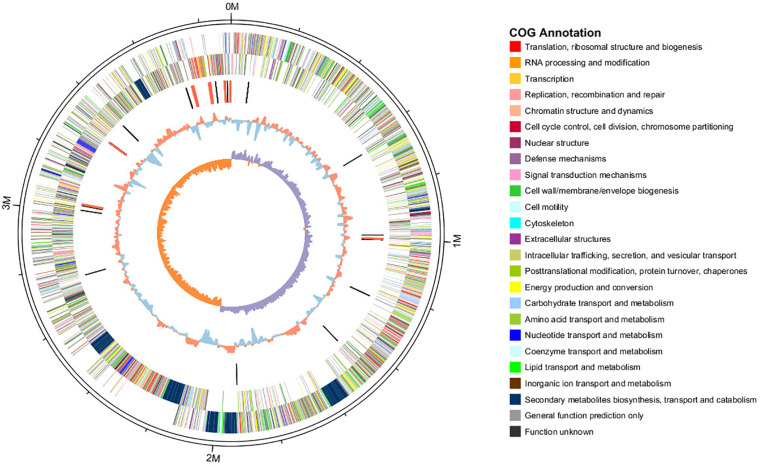
Circular representation of *B. velezensis* HAB-2 genome. From outer to inner circle: first and second circles are positive and negative chain genes, respectively, all genes are color coded according to their COG annotation functions; third circle is ncRNA (black indicates tRNA and red indicates rRNA); fourth circle is GC content (red indicates greater than average value and blue indicates less than average); and fifth circle is GC skew (GC skew = [(G - C)/(G + C), purple indicates > 0 and orange indicates < 0].

**FIGURE 2:**
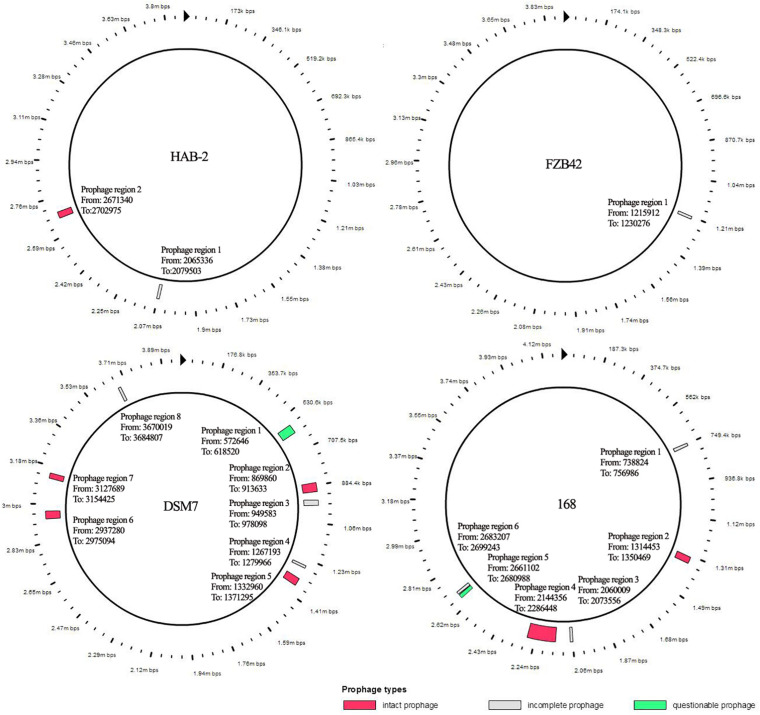
Prophage of *B. velezensis* HAB-2 and comparison with other *Bacillus* spp. All prophage regions in different colors code according to completeness, a prediction of whether the region contains an intact or incomplete prophage. If the region’s total score is < 70, it is marked as incomplete (gray); if within 70–90, it is marked as questionable (green); if > 90, it is marked as intact (red). HAB-2: two prophage regions identified, of which one is intact (Pr 2, with 46 CDS) and one is incomplete (Pr 1, 12 CDS). *Bacillus velezensis* FZB42: one prophage region identified, which is incomplete (Pr 1, 25 CDS). *Bacillus amyloliquefaciens* DSM7: eight prophage regions identified, of which four are intact (Pr2, 46 CDS; Pr 5, 49 CDS; Pr 6, 27 CDS; and Pr 7, 29 CDS), three are incomplete (Pr 3, 24 CDS; Pr 4, 18 CDS; and Pr 8, 17 CDS) and one is questionable (Pr 1, 63 CDS). *Bacillus subtilis* 168: six prophage regions identified, of which two are intact (Pr 2, 49 CDS and Pr 4, 198 CDS), three are incomplete (Pr 1, 20 CDS; Pr 3, 13 CDS; and Pr 6, 23 CDS) and one is questionable (Pr 5, 21 CDS).

The features of the genomes of *Bacillus* reference strains may differ due to the classification of the strains. Thus, we downloaded the genomes of the reference strains from the NCBI database and compared their genomic features with HAB-2. The summary features of the complete genome sequences of HAB-2, FZB42, DSM7 and 168 are summarized in Table 1. There were 143,574 reads with a total of 1,350,725,830 base pairs (bp) in HAB-2, and the sequencing data of HAB-2 were *de novo* assembled using HGAP. Genome circular representation can fully display the characteristics of the genome. We obtained a genome size of approximately 3,894,648 bp, consisting of a circular chromosome and leading to the one scaffold with N50 length of 3.89 Mb and 46.64% GC content (Figure 1). The genome of HAB-2 was smaller than of 168 (4,215,606 bp) and comparable to those of FZB42 (3,918,596 bp) and DSM7 (3,980,199 bp). A total of 3,820 genes were predicted, in which the maximum gene size was 16,302 bp and the minimum was 114 bp. Furthermore, 86 tRNAs and nine sets of 16s-23s-5s ribosomal RNA operons were predicted in the genome (Table 1). The numbers of genes representing the core genome of strains HAB-2, FZB42, DSM7 and 168 were 3820, 3855, 4102, and 4536, respectively (Table 1). The majority of the genes unique to DSM7 and 168 but not present in HAB-2 and FZB42 were phage related (Figure 2). Compared with FZB42, there was one more intact prophage region [Pr 2, with 46 coding sequences (CDS)] in HAB-2 (Figure 2).

**TABLE 1:** Genomic feature comparisons among *B. velezensis* HAB-2, *B. velezensis* FZB42, *B. amyloliquefaciens* DSM7, and *B. subtilis* 168.

	HAB-2	FZB42	DSM7	168
Genome size (bp)	3,894,648	3,918,596	3,980,199	4,215,606
G + C content (mol%)	46.6	46.4	46.1	43.5
Genes	3,820	3,855	4,102	4,536
Percent of coding region	88.8	88.0	86.7	87.2
Ribosomal RNA operons	9	10	10	10
Number of tRNAs	86	89	94	86
Phage-associated genes	58	25	273	324

### Phylogenetic Analysis of *B. velezensis* HAB-2

**FIGURE 3:**
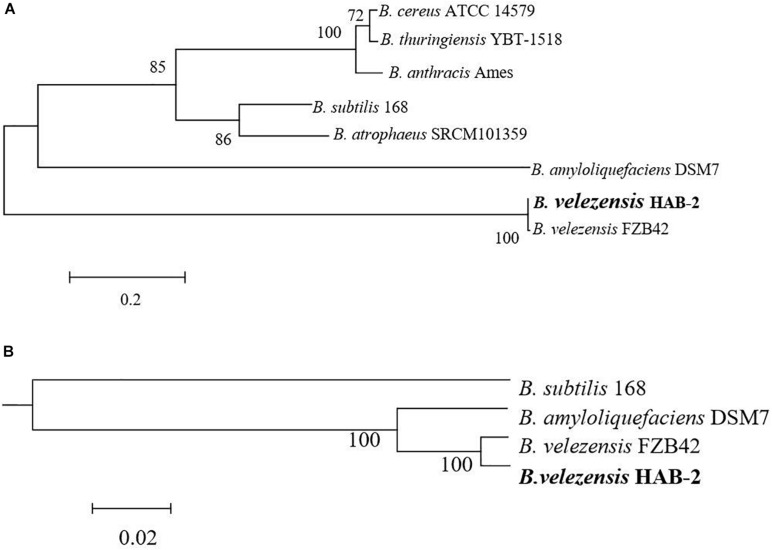
Phylogenetic analysis for *B. velezensis* HAB-2 and reference *Bacillus* strains. **(A)** Phylogenetic tree drawn from the 16S rRNA of *Bacillus* spp. **(B)** Phylogenetic tree drawn from the core genomes of HAB-2, FZB42, DSM7 and 168. Bootstrap values are indicated in% of repetitions.

The classification of *Bacillus* strains has been constantly revised based on the development of molecular technology and the popularity of whole-genome sequencing, especially for *B. velezensis*. A phylogram based on *Bacillus* 16S rRNA, the most useful and is commonly used marker in the systematic classification of bacterium (Coenye and Vandamme, 2010) suggested a close relationship between the genomes of *B. velezensis* HAB-2 and FZB42 (Figure 3A). To further clarify the relationships of HAB-2 with the other relative species, the whole genomic SNPs was used to determine the evolutionary positions of the strains. The phylogenetic tree for HAB-2 and the reference strains was based on genomic scale SNPs (Figure 3B) and showed that HAB-2 was most closely related to *B. velezensis* FZB42. Overall, evolutionary analysis showed that HAB-2 was in the same branch with FZB42.

### Analysis of the Core Genome Among *Bacillus* Species

**FIGURE 4:**
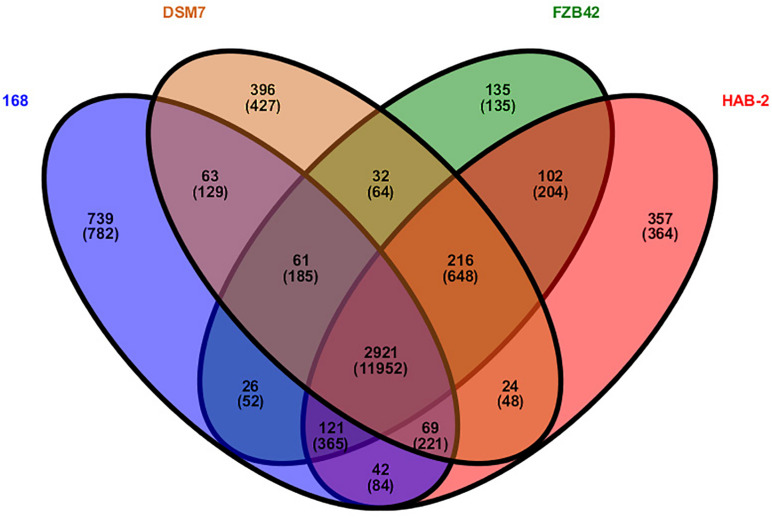
Venn diagram showing the distribution of orthologous gene families in the genomes of *B. velezensis* HAB-2, *B. velezensis* FZB42, *B. amyloliquefaciens* DSM7 and *B. subtilis* 168. This analysis exploits all CDS of the genomes and is not restricted to the core genome. The number above each part represents the number of gene families, while the number below (in parentheses) represents the number of genes.

Research on core genomes is important for determining the functional differences and similarities between strains, and provides molecular evidence for phenotypic differences and similarities. Core-genome plot analyses were performed for the four *Bacillus* genomes (Figure 4). There were 2921 gene families in the final core genome. Among them, 357 (364 CDS), 135 (135 CDS), 396 (427 CDS) and 739 gene families (782 CDS) in HAB-2, FZB42, DSM7, and 168 strains were singletons, respectively. Comparison of homology of the gene families showed that HAB-2 and FZB42 had 3,360 gene families in common with an average 87.22% identity; HAB-2 and DSM7 had 3,230 gene families in common with average 86.84% identity; and HAB-2 and 168 had 3,153 gene families in common with average 81.85% identity. A Venn diagram based on computing of the *Bacillus* gene families suggested high genetic homogeneity among the four *Bacillus* strains and close homology among *B. velezensis* and *B. amyloliquefaciens* genomes.

### Collinearity Analysis of *B. velezensis* HAB-2

**FIGURE 5:**
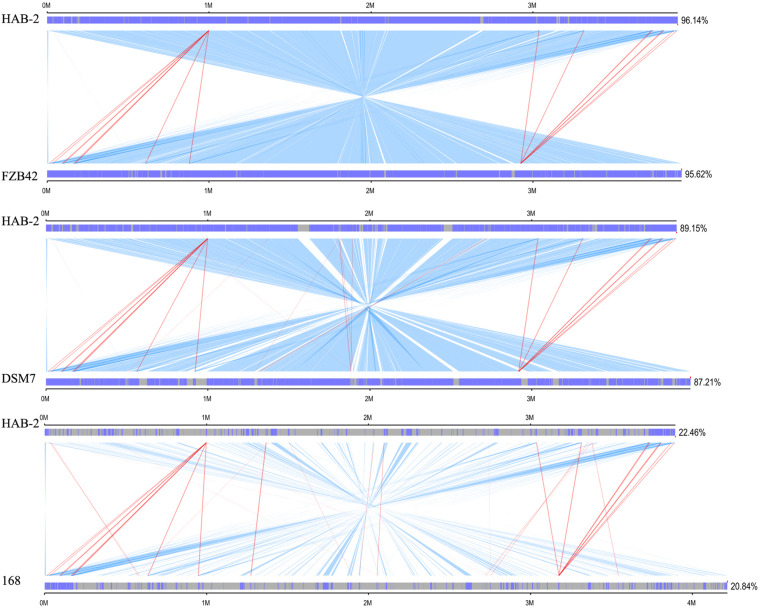
Global alignment of *B. velezensis* HAB-2 and *Bacillus* spp. chromosomes. Indels are depicted by gray rectangles. Dark blue indicates a sequence with synteny, red indicates forward collinearity and light blue indicates negative collinearity.

We performed a collinearity analysis to further compare the genomics similarities and differences among the genome of HAB-2 and the genomes of FZB42, DSM7 and 168. The results showed that the HAB-2 genome displayed different synteny to those of the other strains (Figure 5). The collinearity of the genome of HAB-2 with those of FZB42, DSM7 and 168 represented approximately 96.14, 89.15, and 22.46%, respectively, of the total length of HAB-2. Of all the sequenced genomes, HAB-2 showed the highest synteny with *B. velezensis* FZB42, indicating their evolutionary stages were the closest, and their genomes were more related.

### Gene Clusters Analysis of *B. velezensis* HAB-2

**FIGURE 6:**
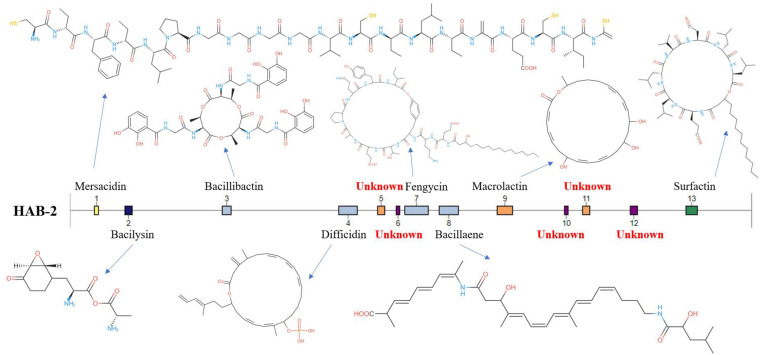
Gene clusters and encoded secondary metabolites identified using the *B. velezensis* HAB-2 genome.

**FIGURE 7:**
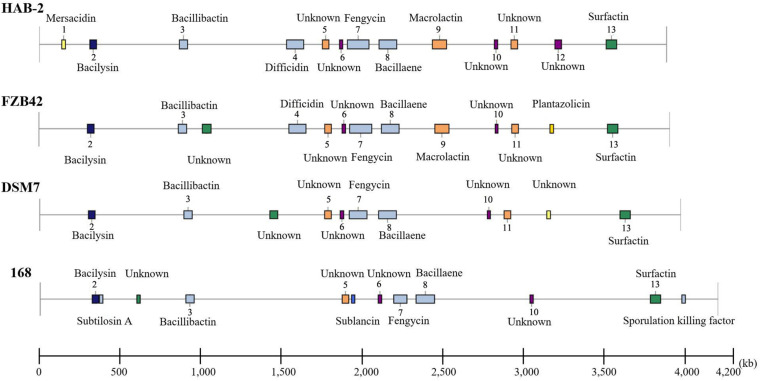
Comparative analysis of the location and products of secondary metabolite gene clusters in *B. velezensis* HAB-2, *B. velezensis* FZB42, *B. amyloliquefaciens* DSM7 and *B. subtilis* 168.

The genomes of *Bacillus* harbor some important gene clusters involved in the synthesis of secondary metabolites, with antimicrobial, antiviral and nematocidal action. Through use of anti-SMASH genome analysis, 13 clusters of secondary metabolites were identified in the HAB-2 genome (Table 2 and Figure 6), covering 20.08% (782.23 kb) of the whole genome and involved in the synthesis of antifungal and antibacterial acting secondary metabolites. This result revealed the potential for biocontrol of plant pathogens in HAB-2. Five gene clusters with a total length of 164.04 kb were shown to direct unknown compound. There were eight gene clusters related to encoding NRPS and PKS, including Type III PKS cluster and PKS-like cluster directing two unknown compounds. Eight clusters were identified as involved in mersacidin synthesis (GenBank AJ250862.2), bacilysin (GenBank CP000560.1), bacillibactin (GenBank AL009126.3), difficidin (GenBank AJ634062.2), fengycin (GenBank CP000560.1), bacillaene (GenBank AJ634060.2), macrolactin (GenBank AJ634061.2) and surfactin (GenBank AJ575642.1) (Table 2 and Figure 6). Secondary metabolites clusters were compared to strains FZB42, DSM7 and 168 (Figure 7). Among the 13 gene clusters, two (Clusters 1 and 12) were specific and only existed in HAB-2, two (Clusters 4 and 9) existed in both HAB-2 and FZB42, one (Cluster 11) existed in three strains excluding 168, and the other eight (Clusters 2, 3, 5–8, 10, and 13) existed in all four strains. Strain HAB-2 had two clusters that were not present in any of the other three strains (Table 2): one (Cluster 1) encoding lanthipeptide was involved in mersacidin synthesis and one (Cluster 12) encoding ladderane was shown to direct an unknown compound. In the same way, we found five clusters (Clusters 5, 6, and 10–12) encoding potential novel metabolites with no previously reported description, and four of these were similar to gene clusters in FZB42.

**TABLE 2:** Comparative analysis of secondary metabolite clusters of *B. velezensis* HAB-2 with *B. velezensis* FZB42, *B. amyloliquefaciens* DSM7, and *B. subtilis* 168,

	HAB-2					Presence (+) or absence (−)		
		
Cluster number	Compound	From	To	Type	Size(kb)	FZB42	DSM7	168
1	Mersacidin	140,027	163,215	Lanthipeptide	23.18	−	−	−
2	Bacilysin	314,409	355,827	Other	41.42	**+**	**+**	**+**
3	Bacillibactin	870,296	920,797	Bacteriocin-nrps	50.50	**+**	**+**	**+**
4	Difficidin	1,536,021	1,641,780	Transatpks	105.76	**+**	−	−
5	Unknown	1,757,688	1,798,401	t3pks	40.71	**+**	**+**	**+**
6	Unknown	1,864,153	1,884,379	Terpene	20.23	**+**	**+**	**+**
7	Fengycin	1,913,038	2,047,159	Transatpks-nrps	134.12	**+**	**+**	**+**
8	Bacillaene	2,109,669	2,219,248	Transatpks-nrps	109.58	**+**	**+**	**+**
9	Macrolactin	2,440,918	2,529,136	Transatpks	88.22	**+**	−	−
10	Unknown	2,825,278	2,846,018	TERPENE	20.74	**+**	**+**	**+**
11	Unknown	2,928,806	2,970,050	pks-like	41.24	**+**	**+**	−
12	Unknown	3,201,340	3,242,461	Ladderane	41.12	−	−	−
13	Surfactin	3,519,488	3,584,895	nrps	65.41	**+**	**+**	**+**

*Secondary metabolite antibacterial gene clusters of the four strains were compared and analyzed using anti-SMASH 4.1.0 (Weber et al., 2015).*

### Biosynthesis and Regulation Genes of Secondary Metabolites

**FIGURE 8:**
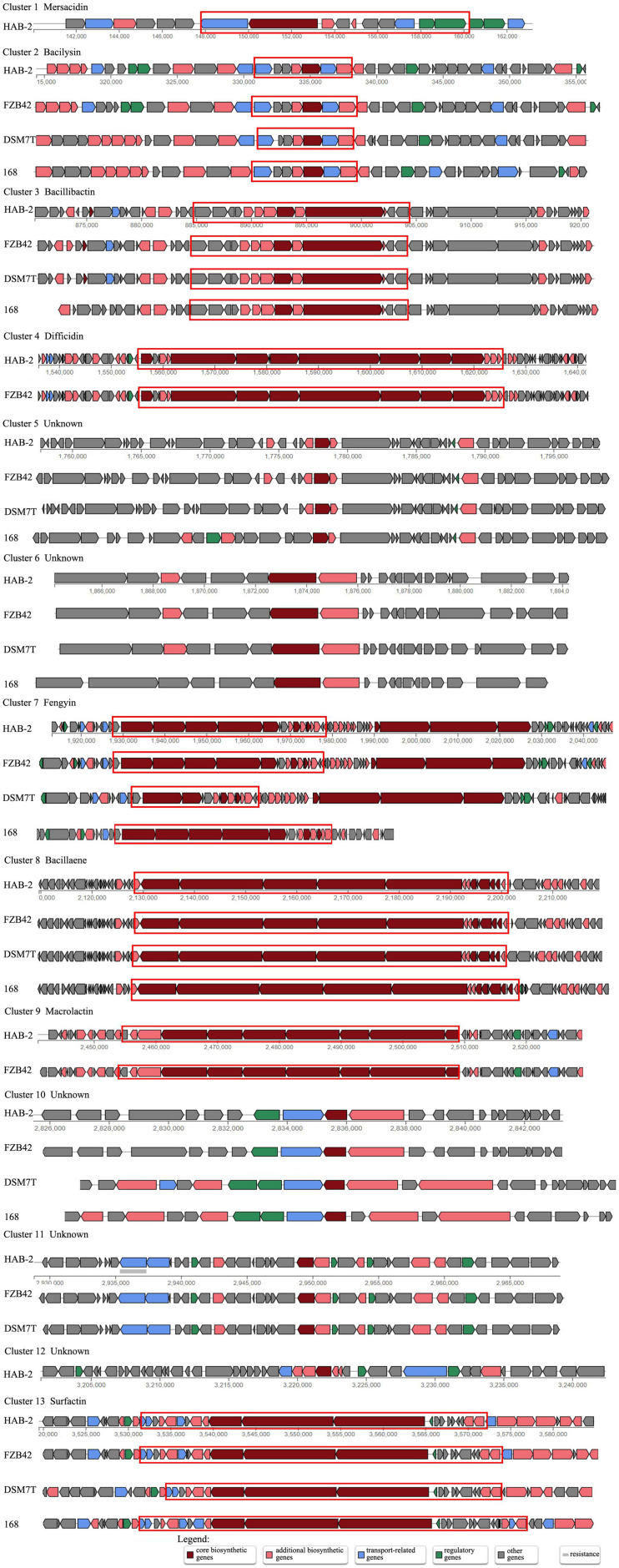
Comparison of biosynthetic gene clusters from *B. velezensis* HAB-2 with *B. velezensis* FZB42, *B. amyloliquefaciens* DSM7 and *B. subtilis* 168. Different genes are filled with different colors: red indicates core biosynthetic genes, pink indicates biosynthetic genes, blue indicates transport-related genes, green indicates regulatory genes and gray indicates other genes. The red circled area is the core biosynthetic part.

Although several gene clusters were shared by HAB-2, FZB42, DSM7, and 168, the gene structures differed (Figure 8). The fengycin cluster lacked three core genes in DSM7: *fenB*, *fenC*, and *fenD*. In the surfactin cluster, HAB-2 lacked *yckE*, DSM7 lacked *ycxC* and *ycxD*, and 168 lacked *yx01* (Table 3). Furthermore, the core biosynthetic genes were similar among the four strains. There was a key gene s*fp* involved in non-ribosomal synthesis, encoding PPTases. A previous study of our research group showed that a *sfp* homolog *lpaH2*, which encoded PPTase, was present in HAB-2 (Jin et al., 2017), was 675 bp in size and encoded 224 amino acids. The Nr annotation information allowed us to obtain the GO function annotation. The GO annotation resulted in three classifications: describing the molecular function (MF), cellular component and biological process (BP) of the gene. Further analysis of the GO annotated genes revealed that *lpaH2* appeared only in MF and BP; and *lpaH2* appeared five times in MF and nine times in BP. Bioinformatics analysis of the GO node was performed on these five and nine branches respectively and showed that the HAB-2_2473 gene was consistent with *lpaH2* function, was 366 bp in size and encoded 120 amino acids. The NCBI alignment showed that HAB-2_2473 had 100% homology with *acpS* and encoded PPTase, a member of the PPTase superfamily. The multiple gene functional bioinformatic methods and resources (including Nr, Swiss-Prot, KOG, KEGG, and Pfam) used to analyze the functions of *lpaH2* and HAB-2_3473 (*acpS*) showed that their functions were highly consistent (Table 4).

**TABLE 3:** Core genes involved with fengycin and surfactin in *B. velezensis* HAB-2, *B. velezensis* FZB42, *B. amyloliquefaciens* DSM7, and *B. subtilis* 168.

Compound	Gene	Presence (+) or absence (−)				Functions or product
		
		HAB-2	FZB42	DSM7	168	
Fengycin	*dacC*	+	+	+	+	DacC protein
	*fenA*	+	+	+	+	Fengycin synthetase A, scaffold biosynthesis
	*fenB*	+	+	−	+	Fengycin synthetase B, scaffold biosynthesis
	*fenC*	+	+	−	+	Fengycin synthetase C, scaffold biosynthesis
	*fenD*	+	+	−	+	Fengycin synthetase D, scaffold biosynthesis
	*fenE*	+	+	+	+	Fengycin synthetase E, scaffold biosynthesis
Surfactin	*yx01*	+	+	+	−	Yx01 protein, alcohol dehydrogenase
	*yckE*	−	+	+	+	YckE protein
	*srfAA*	+	+	+	+	Surfactin synthetase A, scaffold biosynthesis
	*srfAB*	+	+	+	+	Surfactin synthetase B, scaffold biosynthesis
	*srfAC*	+	+	+	+	Surfactin synthetase C, scaffold biosynthesis
	*srfAD*	+	+	+	+	Surfactin synthetase D, other enzymatic
	*aat*	+	+	+	+	Amino transferase
	*ycxC*	+	+	−	+	Transporter
	*ycxD*	+	+	−	+	Transcriptional regulator containing an aminotransferase domain
	*sfp*	+	+	+	+	Phosphopantetheinyl transferase involved in non-ribosomal synthesis
	*yczE*	+	+	+	+	Integral membrane protein involved in non-ribosomal synthesis
	*yckI*	+	+	+	+	YckI protein
	*yckJ*	+	+	+	+	YckJ protein

*Secondary metabolite antibacterial gene clusters of the four strains were compared using anti-SMASH 4.1.0 (Weber et al., 2015), reference gene clusters and core genes analyzed with MIBiG (Minimum Information about a Biosynthetic Gene cluster) (Kautsar et al., 2020).*

**TABLE 4:** Comparison of gene function bioinformatics analysis between *lpaH2* and *acpS.*

	*lpaH2*	*acpS*
Size(bp)	675	366
Nr annotation	4′-Phosphopantetheinyl transferase	4′-Phosphopantetheinyl transferase
Swiss-Prot annotation	4′-Phosphopantetheinyl transferase	Holo-[acyl-carrier-protein] synthase
KOG function description	Phosphopantetheinyl transferase	Phosphopantetheinyl transferase (holo-ACP synthase)
KEGG pathway	Pantothenate and CoA biosynthesis/etabolism of cofactors and vitamins/Metabolism	Pantothenate and CoA Biosynthesis/Metabolism of cofactors and vitamins/Metabolism
PfamA definition	4′-phosphopantetheinyl transferase superfamily	4′-Phosphopantetheinyl Transferase superfamily

## Discussion

This study is surefire to identify and classify *B. velezensis* strain HAB-2 based on genomic information using third-generation sequencing technology. The mechanism of biocontrol of HAB-2 was elucidated by analyzing the gene clusters of secondary metabolites. *Bacillus* species, including *B. velezensis*, *B. amyloliquefaciens*, and *B. subtilis*, have been widely researched because of their potential to produce bacteriostatic secondary metabolites (Fan et al., 2018). *Bacillus velezensis*, isolated from the Vélez River in Málaga in southern Spain, was described in 2005 (Ruiz-García et al., 2005), and *B. velezensis* was later classified as a heterotypic synonym of *B. amyloliquefaciens* (Wang et al., 2008). The model *B. amyloliquefaciens* strain FZB42 was later recognized as synonymous with *B. velezensis* using phylogenomic analysis (Dunlap et al., 2016). The classification of *Bacillus* spp. has become clearer due to the availability of an increasing number of genome sequences and the former *B. amyloliquefaciens* was reclassified and divided into *B. amyloliquefaciens*, *B. siamensis*, and *B. velezensis* (Fan et al., 2017). In this study, we compared genome sequences of reference strains *B. velezensis* FZB42, *B. amyloliquefaciens* DSM7, and *B. subtilis* 168, and conducted phylogenomic analysis; the result showed that HAB-2 was *B. velezensis*, which was previously classified as *B. amyloliquefaciens*.

In this study, we reported a high-quality genome sequence of HAB-2 using third-generation sequencing technology, and made a comparative genome sequence analysis with strains FZB42, DSM7 and 168. The 3.89 Mb genome of HAB-2 encoded 3,820 predicted genes. The four strains were basically identical in regard to whole genome size and the number of genes encoded. The 16S rRNA and genome phylogenetic trees showed high similarity with each other and suggested that HAB-2 was most closely related to FZB42 and DSM7. The differences in the genomes of the four strains were the number and size of prophage regions, and the gene clusters encoding secondary metabolites.

Prophages can constitute 3.1–20% of a bacterium’s genome and are major sources of diversification of bacterial genomes; many prophages appear to be defective and are in a state of mutational decay (Casjens, 2005; Touchon et al., 2016). In our study, the prophage regions of the four strains differed and each strain had a defective prophage. This result also confirmed previous studies. Prophages, including defective ones, can contribute important biological properties to their bacterial hosts, such as expressing virulence genes, to sporulate or to differentiate, and serve as active regulatory switches in bacteria by regulating bacterial genes via genome excision (Casjens, 2005; Feiner et al., 2015). Prophages may indirectly affect the evolvability of bacteria (Touchon et al., 2016). Wang X. et al. (2010) showed that prophages helped the cell respond to various stresses, and inhibiting key proteins encoded by prophages might be a novel means to combat antibiotic resistance. *Pseudomonas aeruginosa* produces three types of bacteriocins, and genetically related phages exist for each type – the gene organization of two types of gene clusters suggested that phage tails have been evolutionarily specialized as bacteriocins (Nakayama et al., 2000). Furthermore, prophages have non-homologous modules that can assemble gene clusters (Casjens, 2005; Gilcrease et al., 2005). Thus, the prophage in *Bacillus* strains may affect the evolutionary, classification and the production of metabolites of the strains.

Based on the *B. velezensis* genomes analysis, more and more secondary metabolite gene clusters have been discovered. *B. velezensis* K26 harbors seven gene clusters and it contains a 1-Deoxynojirumycin biosynthetic gene cluster which is a representative iminosugar with alpha-glucosidase inhibition activity (Lee et al., 2020). *B. velezensis* B-4 harbors 12 gene clusters relating to synthesis of antimicrobial metabolites (Zhu et al., 2020). The HAB-2 genome harbors 13 gene clusters involved in synthesis of antifungal and antibacterial acting secondary metabolites. The number of gene clusters is larger than in other type strains. Notably, HAB-2 has a complete gene cluster, and FZB42 has an incomplete gene cluster, directing mersacidin. Mersacidin is a lanthionine-containing antimicrobial peptide belonging to the family of lantibiotics, and has been identified as a type-B lantibiotic (Bierbaum et al., 1995). It was found in *Bacillus* sp. Strain HIL Y-85,54728 (Chatterjee et al., 1992). People found that mersacidin could act against methicillin-resistant *Staphylococcus aureus* (Brötz et al., 1997, 1998) and was regarded as a lead member of the type-B lantibiotics. Altena et al. (2000) explored the mersacidin biosynthetic gene cluster and reported the complete sequence of a gene cluster of a type-B lantibiotic. Herzner et al. (2011) transferred genomic DNA of a mersacidin-producing strain to FZB42, which resulted in successful production of mersacidin by FZB42.

The GO function annotation showed that gene function was mainly concentrated in MF and BP. Analysis of gene function revealed genes relevant to cellular process, metabolic process, single-organism process, binding and catalytic activity. As previously mentioned, multidomain enzymes (e.g., PKSs and NRPSs) control the biosynthesis of secondary metabolites (Marahiel et al., 1997; Von Dohren et al., 1997; Metz et al., 2001; Cox, 2007). A same common characteristic of these enzymes is that they require post-translational modification to become active. The apoprotein form (inactive) becomes active (holo form) after the covalent attachment of the 4′-phosphopantetheine moiety, which derives from coenzyme A (Lambalot et al., 1996; García-Estrada et al., 2008). This means that this reaction is catalyzed by PPTase. There is a key gene *sfp* encoding the PPTase. Borchert et al. (1994) identified that the *gsp* gene complements a *sfp* deficiency in a *B. subtilis* mutant. Huang et al. (1993) and Tsuge et al. (1996) separately discovered *lpa-14* and *lpa-8*, which regulate production of iturin A, surfactin and plipastatin. Grover et al. (2010) found that *B. subtilis* strain RP24, which possesses antifungal activity, can produce iturin A, surfactin and fengycin and also contains a homolog to *lpa-14* found to encode a PPTase. Morikawa et al. (1992) identified the *B. pumilus* A-1 gene *psf-1*, which regulates surfactin production, and showed that it encoded an active PPTase. Previous study by our research group detected a *sfp* homolog *lpaH2*, which encoded PPTase, in HAB-2 (Jin et al., 2017). The relevant genes encoding PPTases can be screened using a cluster analysis of gene function. In this study, bioinformatics analysis revealed that key gene *acpS*, which encoded 120 amino acids, encoded the PPTases in HAB-2. This provides significant guidance for subsequent analysis of the antibacterial mechanism of *Bacillus* spp.

## Conclusion

We showed the genetic relationship of *B. velezensis* HAB-2 with other *Bacillus* spp. strains through comparative genomic analysis, which is instructive in guiding us to reveal more characteristics of this strain. The differences in prophage regions may provide a new strategy for taxonomic classification and secondary metabolite production of *Bacillus*. The fact that abundant secondary metabolite gene clusters and unique genes exist in the *B. velezensis* HAB-2 genome illustrates that this strain has potential as a biocontrol bacterium for plant protection in agriculture.

## Data Availability Statement

The datasets presented in this study can be found in online repositories. The names of the repository/repositories and accession number(s) can be found below: https://www.ncbi.nlm. nih.gov/genbank/, MT375545; https://www.ncbi.nlm.nih. gov/genbank/, MT386600; https://www.ncbi.nlm.nih.gov/, CP060085.

## Author Contributions

WM and PX designed the experiments and wrote the manuscript. PX, SX, WL, and PJ performed collection and bioinformatics analysis. SX, DW, DY, and YW revised the manuscript. All authors read and approved the final manuscript.

## Conflict of Interest

The authors declare that the research was conducted in the absence of any commercial or financial relationships that could be construed as a potential conflict of interest.

## Footnotes

1 http://www.chinapesticide.org.cn/

2 http://phast.wishartlab.com/

3 ftp://ftp.ebi.ac.uk/pub/databases/Pfam/Tools/PfamScan.tar.gz
